# HDAC Class I Inhibitor Domatinostat Preferentially Targets Glioma Stem Cells over Their Differentiated Progeny

**DOI:** 10.3390/ijms23158084

**Published:** 2022-07-22

**Authors:** Yurika Nakagawa-Saito, Shinichi Saitoh, Yuta Mitobe, Asuka Sugai, Keita Togashi, Shuhei Suzuki, Chifumi Kitanaka, Masashi Okada

**Affiliations:** 1 Department of Molecular Cancer Science, School of Medicine, Yamagata University, 2-2-2 Iida-nishi, Yamagata 990-9585, Japan; yrk@med.id.yamagata-u.ac.jp (Y.N.-S.); y-mitobe@med.id.yamagata-u.ac.jp (Y.M.); s-asuka@med.id.yamagata-u.ac.jp (A.S.); ke-togashi@med.id.yamagata-u.ac.jp (K.T.); s-suzuki@med.id.yamagata-u.ac.jp (S.S.); 2 Department of Immunology, School of Medicine, Yamagata University, 2-2-2 Iida-nishi, Yamagata 990-9585, Japan; s-saitoh@med.id.yamagata-u.ac.jp; 3 Department of Neurosurgery, School of Medicine, Yamagata University, 2-2-2 Iida-nishi, Yamagata 990-9585, Japan; 4 Department of Ophthalmology and Visual Sciences, School of Medicine, Yamagata University, 2-2-2 Iida-nishi, Yamagata 990-9585, Japan; 5 Department of Clinical Oncology, School of Medicine, Yamagata University, 2-2-2 Iida-nishi, Yamagata 990-9585, Japan; 6 Research Institute for Promotion of Medical Sciences, Faculty of Medicine, Yamagata University, 2-2-2 Iida-nishi, Yamagata 990-9585, Japan

**Keywords:** glioma initiating cell, tumor initiating cell, epigenetic modulation, stemness

## Abstract

Cancer stem cells (CSCs) are in general characterized by higher resistance to cell death and cancer therapies than non-stem differentiated cancer cells. However, we and others have recently revealed using glioma stem cells (GSCs) as a model that, unexpectedly, CSCs have specific vulnerabilities that make them more sensitive to certain drugs compared with their differentiated counterparts. We aimed in this study to discover novel drugs targeting such Achilles’ heels of GSCs as anti-GSC drug candidates to be used for the treatment of glioblastoma, the most therapy-resistant form of brain tumors. Here we report that domatinostat (4SC-202), a class I HDAC inhibitor, is one such candidate. At concentrations where it showed no or minimal growth inhibitory effect on differentiated GSCs and normal cells, domatinostat effectively inhibited the growth of GSCs mainly by inducing apoptosis. Furthermore, GSCs that survived domatinostat treatment lost their self-renewal capacity. These results suggested that domatinostat is a unique drug that selectively eliminates GSCs not only physically by inducing cell death but also functionally by inhibiting their self-renewal. Our findings also imply that class I HDACs and/or LSD1, another target of domatinostat, may possibly have a specific role in the maintenance of GSCs and therefore could be an attractive target in the development of anti-GSC therapies.

## 1. Introduction

Glioblastoma (GBM), the most malignant form of glioma and the most common primary brain malignancy in adults, remains among the most aggressive of all human cancers. The standard treatment for GBM consists of surgical resection followed by chemoradiotherapy. However, even after optimal treatment, disease progression including local and/or distant recurrence is almost inevitable, which is reflected in the dismal 5-year survival rate (~10% at best) of GBM [1,2].

Cancer stem cells (CSCs), a small subpopulation of undifferentiated tumor cells endowed with self-renewal and tumor-initiating capacities, give rise to differentiated progeny (non-CSCs) that contribute to the heterogeneity of tumor tissues and sustain the growth of tumors. Similar to normal stem cells, CSCs are characterized by their higher capacity than non-CSCs to survive stressful conditions, which renders them resistant to conventional cancer therapies. As such, CSCs survive, for instance, chemotherapy and/or radiotherapy to initiate tumors and thus play a key role in tumor recurrence after apparently successful initial treatment [3,4,5]. Glioma stem cells (GSCs), the CSCs of GBM, are therefore regarded as a potential therapeutic target to prevent recurrence and achieve long-term survival of patients with GBM.

While CSCs are in general resistant to cell death compared with non-CSCs [3,4,5], emerging evidence now suggests that CSCs have unique vulnerabilities for which they show higher sensitivity to certain types of drugs than their differentiated progeny. We previously demonstrated using GSC lines and their isogenic, differentiated counterparts that licochalcone A, a herbal medicine with known inhibitory activity against complex III of the mitochondrial electron transport chain, is selectively cytotoxic to GSCs [6], which led to the subsequent discovery that GSCs are characterized by increased oxidative phosphorylation, which they depend on heavily for survival, and can be targeted with verteporfin, a drug approved for the treatment of macular degeneration [7]. We also found recently that methotrexate, an antimetabolite which has long been used to treat a variety of human cancers, exerts preferential cytotoxicity toward GSCs over their differentiated counterparts due to their dependence on folate metabolism as represented by increased expression of the reduced folate carrier RFC-1/SLC19A1 in GSCs [8]. This is in line with the reported dependence of GSCs on purine synthesis [9] and together suggests that folate-dependent one-carbon metabolism involved in purine synthesis may be one of the vulnerabilities of GSCs. Thus, these lines of evidence reinforce the idea that GSCs have “druggable Achilles’ heels” that could be targeted to treat GSCs. In an attempt to bring to light such Achilles’ heels of GSCs and to exploit them to develop therapies targeting GSCs, we have been seeking to find drugs that preferentially inhibit the growth of GSCs compared with their differentiated counterparts.

Inhibitors targeting one or more of histone deacetylases (HDACs) are known to elicit anticancer effects. In human, 18 HDACs are subdivided into four classes based on their amino acid sequences. Class I (HDACs 1–3 and 8), class IIa (HDACs 4, 5, 7, and 9), class IIb (HDACs 6 and 10), and class IV (HDAC11) are zinc-dependent metallohydrolases, whereas class III HDACs (sirtuins 1–7) are NAD^+^-dependent [10]. Domatinostat (4SC-202), a new class I HDAC inhibitor, is among such HDAC inhibitors developed as an anticancer agent [11,12,13]. Here in this study, we identified domatinostat as a drug that preferentially inhibits the growth of GSCs relative to their differentiated counterparts. Further analysis revealed that domatinostat not only inhibits the proliferation and survival of GSCs but also causes loss of their CSC properties, highlighting the potential of domatinostat as an anti-GSC drug.

## 2. Results

### 2.1. Preferential Inhibition of Glioma Stem Cell Growth by Domatinostat

**Figure 1:**
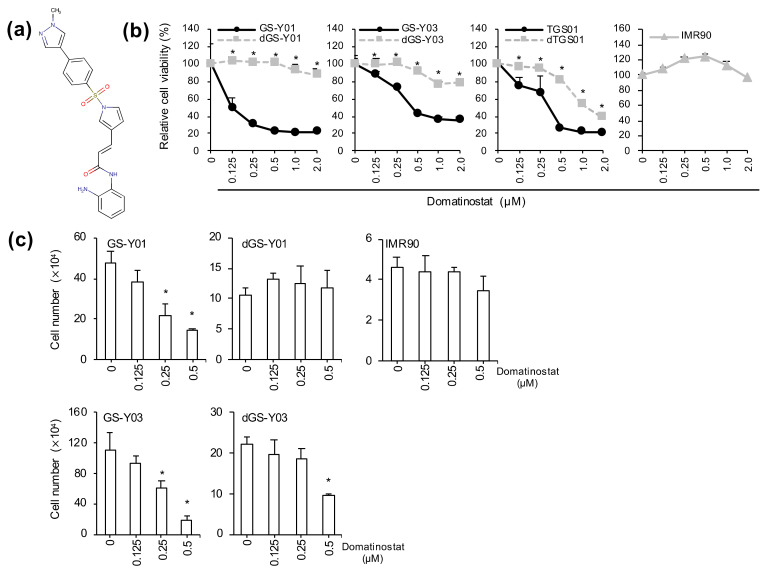
Effect of domatinostat on the growth of glioma stem cell lines, their differentiated counterparts, and normal fibroblasts. Values are presented as the means + SD of the triplicate samples. Similar results were obtained from two independent biological replicates. (**a**) The chemical structure of domatinostat (4SC-202). (**b**) Determination of metabolic viability. GS-Y01, GS-Y03, TGS01, their differentiated counterparts (dGS-Y01, dGS-Y03, dTGS01), and IMR90 human lung fibroblasts treated with the indicated concentrations of domatinostat for 3 days were subjected to the WST assay. * *p* < 0.05 vs. undifferentiated glioma stem cells treated at the same concentration by the Student’s *t*-test. (**c**) Viable cell count determined by dye exclusion. GS-Y01, GS-Y03, their differentiated counterparts (dGS-Y01, dGS-Y03), and IMR90 human lung fibroblasts were treated with the indicated concentrations of domatinostat for 3 days, and the number of viable cells was determined by trypan blue dye exclusion. * *p* < 0.05 vs. cells treated without domatinostat (i.e., at 0 μM) by the Student’s *t*-test.

In the course of screening drugs of interest, we tested domatinostat (Figure 1a), a small molecule epigenetic modulator in clinical development, on pairs of GSCs and their differentiated isogenic counterparts and determined its effect on cell growth. The results of the WST assay indicated that the reduction in cell viability after domatinostat treatment was more pronounced in GSCs, suggesting that domatinostat may have preferential growth inhibitory effects on GSCs over differentiated GSCs (Figure 1b). To determine whether the reduction in metabolic activity observed in the WST assay actually reflected a reduction in the number of viable cells, a trypan blue exclusion test was performed, which demonstrated that domatinostat inhibited the growth of GSCs more effectively than that of their differentiated counterparts (Figure 1c). Importantly, we also confirmed that the growth-inhibitory effect of domatinostat on GSCs was evident at concentrations where it did not show any growth inhibitory effects on IMR90 human lung fibroblasts (Figure 1b,c).

**Figure 2:**
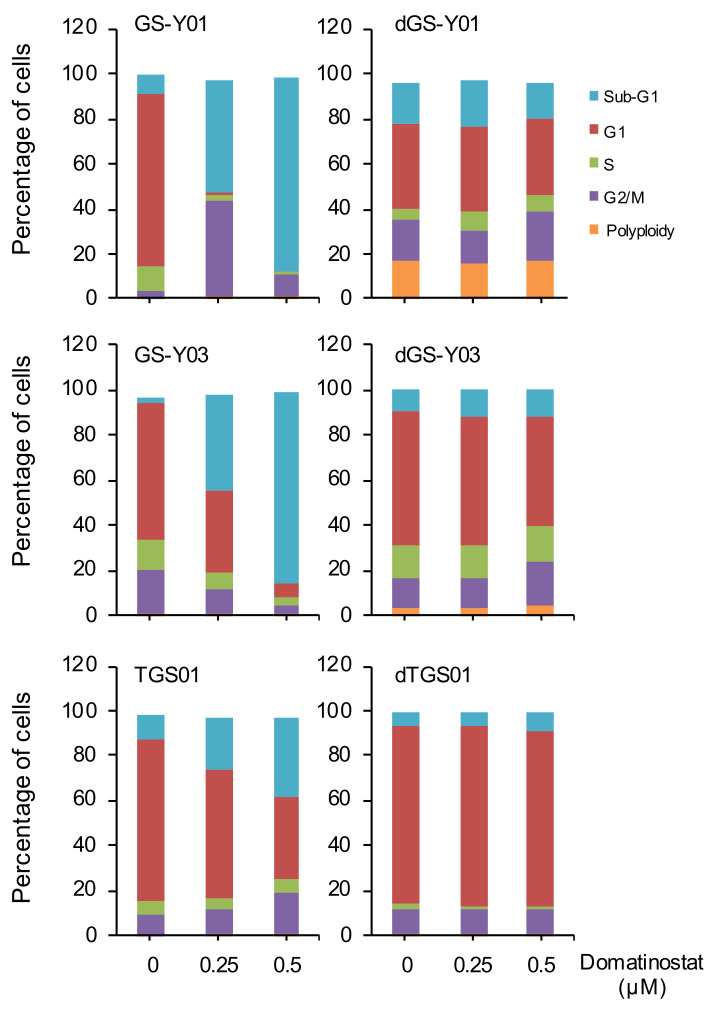
Effect of domatinostat on the cell-cycle distribution of glioma stem cell lines and their differentiated counterparts. GS-Y01, GS-Y03, TGS01, and their differentiated counterparts (dGS-Y01, dGS-Y03, and dTGS01) treated with the indicated concentrations of domatinostat for 3 days were subjected to flow cytometric analysis of cellular DNA content by propidium iodide staining. Results from a representative experiment repeated in two independent biological replicates with similar results are shown.

To elucidate the cellular mechanisms underlying the growth inhibitory effects of domatinostat on GSCs, we next analyzed the impact of domatinostat treatment on the cell cycle distribution of GSCs and their differentiated counterparts (Figure 2). Remarkably, domatinostat treatment caused a dramatic increase in the sub-G1 population in GS-Y01 and GS-Y03, suggesting that domatinostat induced massive DNA fragmentation in these GSCs. Notably, while domatinostat caused a significant, though not as pronounced as in GS-Y01 and GS-Y03, increase in the sub-G1 population in TGS01, it also increased the G2/M population at the same time, in line with earlier reports that domatinostat blocked cell cycle progression at the G2/M transition in human cancer cells [14,15,16]. These results suggested that domatinostat may induce either DNA fragmentation in GSCs or cell cycle arrest at the G2/M border in intact GSCs. Importantly, domatinostat caused no significant changes in the cell cycle distribution of differentiated cells, consistent with its lack of growth inhibitory effects on these cells (Figure 2). Together, the results suggested that domatinostat may inhibit the growth of GSCs by inducing cell death accompanied by DNA fragmentation and/or cell cycle arrest at G2/M.

### 2.2. Domatinostat Activates the Caspase Pathway and Induces Cell Death in GSCs

**Figure 3:**
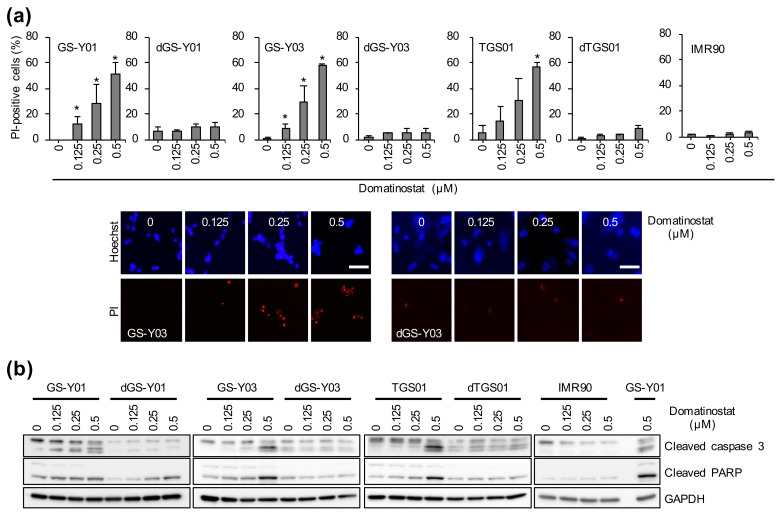
Induction of cell death and apoptotic caspase activation by domatinostat. (**a**) Induction of cell death. GS-Y01, GS-Y03, TGS01, their differentiated counterparts (dGS-Y01, dGS-Y03, dTGS01), and IMR90 human lung fibroblasts treated with the indicated concentrations of domatinostat for 3 days were subjected to the propidium iodide (PI) incorporation assay. Values are presented as the means + SD of triplicate samples. * *p* < 0.05 vs. cells treated without domatinostat (i.e., at 0 μM) by the Student’s *t*-test. Representative fluorescence images of Hoechst- (upper rows) and PI- (lower rows) positive cells are also shown. Bars: 50 μm. (**b**) Activation of the caspase pathway. The indicated glioma stem cells (GS-Y01, GS-Y03, and TGS01), their differentiated counterparts (dGS-Y01, dGS-Y03, and TGS01), and IMR90 human lung fibroblasts were treated with the indicated concentrations of domatinostat for 3 days and then subjected to Western blot analysis for the expression of the indicated proteins.

The increase in the sub-G1 population after domatinostat treatment suggested that domatinostat may induce cell death accompanied by DNA fragmentation, most likely apoptosis, in GSCs. Indeed, the results of the propidium iodide (PI) incorporation assay indicated that the percentage of dead cells increased with increasing concentrations of domatinostat in GSCs but not in differentiated GSCs or normal fibroblasts (Figure 3a). Notably, the expression levels of cleaved caspase 3 and cleaved PARP increased just in parallel with the increase in the percentage of dead cells (Figure 3b), demonstrating the activation of the caspase pathway indicative of apoptosis during domatinostat-induced cell death. Together, these results suggested that domatinostat may have GSC-specific cytotoxicity via apoptosis induction.

### 2.3. Self-Renewal Capacity Is Lost in GSCs That Survived Domatinostat Treatment

**Figure 4:**
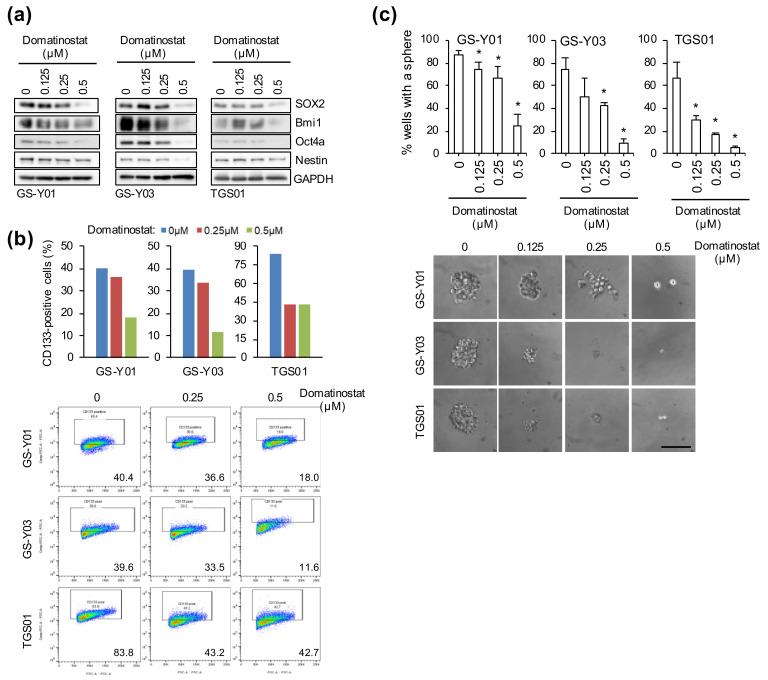
Effect of domatinostat on the self-renewal capacity of glioma stem cells. (**a**,**b**) Stem cell marker expression. Cells treated with the indicated concentrations of domatinostat for 3 days were subjected to Western blot analysis of the expression of the indicated proteins (**a**), or to flow cytometric analysis of CD133 expression on the cell surface to determine the percentage of CD133-positive cells (**b**). Representative flow cytometric plots together with the percentage of CD133-positive cells are also shown in (**b**). (**c**) Sphere formation assay. GS-Y01, GS-Y03, and TGS01 cells treated with domatinostat at the indicated concentrations for 3 days were subjected to the sphere formation assay in the absence of domatinostat. Top: percentage of wells in which a tumorsphere was formed from a single cell. * *p* < 0.05 vs. cells treated without domatinostat (i.e., at 0 μM) by the Student’s *t-*test. Bottom: photomicrographs of representative wells. Bar: 100 μm.

We were next interested in investigating the impact of domatinostat treatment on the CSC properties of GSCs. To this end, we first examined by Western blot analysis the expression levels of nuclear (SOX2, Bmi1, and Oct4a) and cytoplasmic (Nestin) stem cell markers in GSCs treated with domatinostat. Domatinostat caused a uniform decrease in the expression levels of these stem cell markers (Figure 4a). We also conducted a flow cytometric analysis of the expression of a cell-surface stem cell marker CD133. Again, we found that the proportion of CD133-positive cells decreased with domatinostat treatment in parallel with the nuclear and cytoplasmic stem cell markers (Figure 4b). These results strongly suggested that stem cell properties were being lost in GSCs after domatinostat treatment. To test this idea, we evaluated the ability of GSCs to self-renew as spheres in the absence of domatinostat after domatinostat pretreatment. The results of the sphere formation assay clearly indicated that domatinostat pretreatment inhibited the ability of GSCs to form spheres even in the absence of domatinostat (Figure 4c). Collectively, these results suggested that domatinostat impaired the self-renewal capacity of GSCs.

## 3. Discussion

We have shown in this study that domatinostat exhibits preferential inhibitory activity against GSCs over their differentiated counterparts. Not only did domatinostat interfere with the survival of GSCs at concentrations where it did not compromise the growth of differentiated GSCs or normal fibroblasts, it also effectively impaired the self-renewal capacity of GSCs. Our findings suggest that domatinostat may be more of an anti-GSC drug than a drug targeting glioma cells in general, which needs to be taken into consideration in the clinical application of domatinostat for the treatment of glioma patients.

With the increasing interest in HDAC inhibitors as potential cancer therapeutics, the anti-cancer activity of domatinostat, a novel class I-selective HDAC inhibitor, has been actively explored in the past several years and has been demonstrated in cancer cells from solid tumors as well as hematological malignancies. Interestingly, while earlier studies tested domatinostat mostly using conventional cancer cell lines [11,12,13,14,15,16,17,18,19,20], focus has been directed to its effects on CSCs in recent studies [21,22,23,24]. The results of the recent studies suggested the possibility that CSCs are the favored target of domatinostat, yet this possibility has not been formally tested and therefore remains to be demonstrated conclusively. In this regard, our study clearly demonstrated that, using pairs of differentiated and undifferentiated GSC lines, the growth inhibitory effects of domatinostat were more pronounced in undifferentiated GSCs and that it even impaired the most important properties of GSCs, namely the self-renewal capacity. The molecular mechanisms accountable for the differential effects of domatinostat are currently unclear. Differential expression of class I HDACs, the *bona fide* targets of domatinostat, in differentiated and undifferentiated GSCs could be a possible explanation. However, this was unlikely to be the case as there were no significant differences in the expression levels of HDACs 1 and 2 between undifferentiated and differentiated GSCs. Although there was a trend toward increased expression of HDAC3 in undifferentiated GSCs, the results were not consistent across cell lines (Y. N.-S. and M. O., unpublished data). Additionally of interest in this regard was a recent report that domatinostat reduced the protein levels of FOXM1 in pancreatic cancer cells [23], which reportedly plays a crucial role in the maintenance of GSCs [25]. However, although domatinostat did reduce the expression of FOXM1 in GSCs, our preliminary data also indicated that the knockdown of FOXM1 in GSCs alone failed to mimic the effects of domatinostat on GSCs (Y. N.-S. and M. O., unpublished data), suggesting that at least mechanisms other than those involving FOXM1 are at play.

With the accumulating evidence that their expression is deregulated in glioma, HDACs have drawn increasing attention as promising therapeutic targets and, accordingly, inhibitors targeting HDACs have been tested in many preclinical and clinical studies of glioma [26,27,28]. However, none of the tested HDAC inhibitors have proven beneficial so far in patients with glioma, except for valproic acid tested in a phase 2 study in combination with temozolomide and radiotherapy [26,27]. There could be a number of potential reasons for their failure to show activity in glioma patients, among which is the high toxicity and low specificity of the HDAC inhibitors used [29]. To date, 18 HDACs have been identified and are categorized into four classes (I through IV). To overcome the issues arising from the lack of selectivity of classical broad-spectrum HDAC inhibitors that target multiple classes of HDACs (such as trichostatin A and valproic acid), the development of class- or isoform-selective HDAC inhibitors have been actively sought [29,30]. Domatinostat (4SC-202), whose effects on glioma cells we tested in the present study, is one such class-selective HDAC inhibitor that selectively targets class I HDACs [11,12,13]. Setting aside romidepsin/depsipeptide/FK228, whose selectivity as a class I-selective HDAC inhibitor is called into question [31], among known class I-selective HDAC inhibitors, entinostat (MS-275) has shown growth inhibitory activity against glioma cells so far, albeit in combination with chemotherapeutic agents or molecular targeting drugs [32,33]. Here in this study, we have successfully demonstrated that domatinostat is, to the best of our knowledge, the first class I-selective HDAC inhibitor capable of targeting GSCs alone, which may imply that class I HDACs have essential roles in GSCs and therefore could be viable molecular targets in developing novel therapies aimed at GSCs. However, since domatinostat is capable of inhibiting lysine-specific demethylase 1 (LSD1), which demethylates lysine residues of histone H3K4me1/2 and H3K9me1/2 at clinically relevant concentrations in addition to class I HDACs [12], not only class I HDACs but also LSD1 may comprise a selective vulnerability (Achilles’ heel) of GSCs.

Although the clinical benefit of domatinostat in cancer patients is yet to be determined, a phase 1 study was conducted in patients with advanced hematological malignancies to determine the safety, tolerability, pharmacokinetics, pharmacodynamics, and antitumor activity of domatinostat [34]. The results of the study were promising, demonstrating that administration of domatinostat was safe and well tolerated with signs of antitumor activity. Significantly, the results of the pharmacokinetic analysis indicate that the concentration range of domatinostat required to show anti-GSC effects in the present study (~500 nM) is clinically achievable. In considering clinical application of domatinostat for the treatment of brain tumors, it is also of interest whether domatinostat crosses the blood–brain barrier (BBB), at which efflux transporters excrete anticancer drugs and tight junctions between the capillary endothelial cells block their entry [35]. There is no data currently available as to the BBB penetrability of domatinostat. However, since the BBB is known to be disrupted in brain tumors [36,37], domatinostat is expected to at least cross the BBB and reach tumor cells present in tumor masses. If domatinostat is to be delivered across the BBB, it might be an attractive approach, for instance, to conjugate domatinostat with a relevant antibody so that it may cross the BBB via receptor-mediated transcytosis [35]. In terms of clinical application, future preclinical animal studies are also desired to confirm whether the pharmacodynamic profiles of domatinostat delineated in vitro in this study are maintained in vivo. If domatinostat selectively targets GSCs in vivo, it will effectively inhibit the formation of new tumors (from implanted GSCs) but not the growth of established tumors, which is driven primarily by the proliferation of non-GSC tumor cells. Such preclinical data will help guide rational use of domatinostat in the treatment of patients with glioma; proper combination with conventional therapies targeting “non-GSC” tumor cells might be key to maximizing the therapeutic potential of domatinostat. Although it remains to be shown whether other HDAC inhibitors share the CSC-specific activity of domatinostat, if indeed they do, it could be one of the unrecognized reasons why HDAC inhibitors appear to do better as part of a combination therapy than as a monotherapy [26].

In summary, we demonstrated for the first time that domatinostat exhibits anti-cancer activity in glioma cells, more specifically, GSCs. The ability of domatinostat to effectively induce cell death and impair the self-renewal capacity of GSCs at the same time makes it a promising candidate for use in the treatment of GBM to prevent recurrence arising from residual GSCs after conventional therapies.

## 4. Materials and Methods

### 4.1. Reagents and Antibodies

Domatinostat (4SC-202; S7555) was purchased from Selleck Chemicals (Houston, TX, USA). Domatinostat was dissolved in DMSO to prepare a 10 mM stock solution. The structure of domatinostat was obtained from DrugBank [38]. Propidium iodide (P3566) and Hoechst33342 (H3570) solutions were purchased from Thermo Fisher Scientific, (Waltham, MA, USA). Trypan blue solution (T8154) was purchased form Merck (Darmstadt, Germany). Antibodies against Bmi1 (05-637) and Nestin (MAB5326) were purchased from Merck. An antibody against SOX2 (MAB2018) was purchased from R&D Systems Inc. (Minneapolis, MN, USA). Anti-CD133 (W6B3C1) was purchased from Miltenyi Biotech (Bergisch Gladbach, Germany). Antibodies against, OCT-4a (#2890), GAPDH (#5174), cleaved PARP (#9541), and cleaved caspase 3 (#9661) were purchased from Cell Signaling Technology Inc. (Beverly, MA, USA).

### 4.2. Cell Culture

The human GSCs used in this study (GS-Y01, GS-Y03, and TGS01) were maintained under previously reported monolayer stem cell culture conditions [8,39]. The differentiation of GSCs was induced by culturing cells in a DMEM/F-12 medium supplemented with 10% fetal bovine serum (FBS), 100 units/mL of penicillin, and 100 μg/mL of streptomycin for 2 weeks [8,39]. IMR90, a human normal fetal lung fibroblast cell line, was obtained from the American Type Culture Collection (Manassas, VA, USA) and maintained in DMEM supplemented with 10% FBS. All IMR90 experiments were performed using cells with a low passage number (<8).

### 4.3. Cell Viability Assay

Cell viability was evaluated using the WST-8 assay [7,8]. Cells (0.5–1 × 10^4^/well) plated on 96-well collagen I-coated plates (GSCs) or non-coated plates (differentiated GSCs and IMR90) were treated with domatinostat, as described in the figure legends. The WST-8 reagent (Cell Counting Kit-8, DOJINDO LABORATORIES, Kumamoto, Japan) was then added and cells were incubated at 37 °C for 1–3 h. Absorbance at 450 nm was measured using a microplate reader (iMark; Bio-Rad, Hercules, CA, USA). Relative cell viability was calculated as a percentage of the absorbance of treated samples relative to that of controls.

### 4.4. Trypan Blue Dye Exclusion Assay

The numbers of viable and dead cells were determined using trypan blue dye exclusion assay [40,41]. Cells treated with domatinostat as described were pipetted (GSCs) or trypsinized (differentiated GSCs and IMR90) and suspended in phosphate-buffered saline (PBS), and then cells were stained with 0.2% trypan blue for 1 min. Viable and dead cells were identified by their ability and inability, respectively, to exclude trypan blue using a hemocytometer.

### 4.5. Propidium Iodide Incorporation Assay

To assess cell death, the propidium iodide (PI) incorporation assay was used [40,41]. Cells treated with domatinostat as indicated in the figure legends were incubated with PI (1 μg/mL) and Hoechst33342 (10 μg/mL) for 5 min at 37 °C. To calculate the ratio of PI-positive cells (dead cells) to Hoechst-positive cells (total cells), fluorescent images were obtained using a fluorescence microscope (CKX41; Olympus, Tokyo, Japan) equipped with iPhone and scored. More than 170 cells were counted to calculate the percentage of PI-positive cells.

### 4.6. Flow Cytometric Analysis

Cell surface expression of CD133 was assessed using flow cytometric analysis [40,42]. The dissociated cells were washed with PBS, fixed with 4% (*w/v*) paraformaldehyde at room temperature (RT) for 10 min, and washed again with PBS. Cells were blocked in FCM buffer (0.5% (*w/v*) bovine serum albumin and 0.1% (*w/v*) NaN_3_ in PBS) for 1 h, followed by three PBS rinses, a further incubation with the anti-CD133 antibody in FACS buffer at 4 °C overnight, and then an incubation with Alexa Fluor^®^ 488 goat anti-mouse IgG at RT for 1 h in the dark. Cells exhibiting a signal for CD133 above the gate established by the isotype control were considered to be positive for CD133. At least 1 × 10^4^ cells were evaluated and gated using side and forward scatters to identify viable cell populations.

Cell cycle status was analyzed by the standard PI staining protocol using flow cytometry [43,44]. Cells treated with domatinostat were suspended with ice-cold PBS and fixed in 70% ethanol. Tubes containing the cell pellets were stored at −20 °C. The fixed cells were washed with ice-cold PBS three times and treated with RNase A (20 ug/mL) for 30 min at 37 °C, and then an incubation with PI (20 μg/mL). After incubation, at least 1 × 10^4^ cells were analyzed. All flow cytometry experiments were run on the FACSMelody^TM^ flow cytometer (BD Biosciences, Franklin Lakes, NJ, USA) and data were analyzed using FlowJo software, version 10.8.1 (FlowJo LLC, Ashland, OR, USA).

### 4.7. Western Blot Analysis

Western blot analysis was conducted as previously described [8,45]. Cells were harvested and washed with ice-cold PBS and lysed in RIPA buffer (10 mM Tris/HCl (pH 7.4), 0.1% sodium dodecyl sulfate (SDS), 0.1% sodium deoxycholate, 1% Nonidet P-40, 150 mM NaCl, 1 mM EDTA, 1.5 mM sodium orthovanadate, 10 mM sodium fluoride, 10 mM sodium pyrophosphate, and protease inhibitor cocktail set III (FUJIFILM Wako Chemicals, Osaka, Japan)). The lysates were immediately mixed with the same volume of 2 × Laemmli buffer (125 mM Tris/HCl (pH 6.8), 4% SDS, and 10% glycerol) and boiled at 95 °C for 10 min. After the protein concentrations of the cell lysates were measured using a BCA protein assay kit (Pierce Biotechnology, Inc., Rockford, IL, USA), samples containing equal amounts of protein were separated by SDS/polyacrylamide gel electrophoresis and transferred to polyvinylidene difluoride membranes. Membranes were probed with the indicated primary antibodies followed by appropriate HRP-conjugated secondary antibodies as recommended by the manufacturer of each antibody. Immunoreactive bands were visualized using Immobilon Western Chemiluminescent HRP Substrate (Merck Millipore, Burlington, MA, USA) and detected by a ChemiDoc Touch device (Bio-Rad).

### 4.8. Sphere Formation Assay

The sphere formation assay was performed as previously described [40,42]. Cells in the monolayer culture were dissociated, serially diluted in the stem cell culture medium, and then seeded onto non-coated 96-well plates such that each well contained a single cell. Wells containing a single cell were marked under a phase-contrast microscope the next day, and the percentage of marked wells with a sphere relative to the total number of marked wells was calculated 7–10 days after seeding.

### 4.9. Statistical Analysis

Results are shown as means + standard deviations (SD). Data were analyzed using the Student’s *t*-test for comparisons between two groups. Differences with a *p*-value <0.05 were considered to be significant and are indicated with asterisks in the figures.

## Data Availability

All data are contained in this article and there are no repository data.
